# The Relationship between Serum Uric Acid Level and Metabolic Syndrome: Differences by Sex and Age in Taiwanese

**DOI:** 10.2188/jea.JE20090078

**Published:** 2010-05-05

**Authors:** Wen-Ko Chiou, Ming-Hsu Wang, Ding-Hau Huang, Hsin-Tzu Chiu, Yun-Ju Lee, Jen-Der Lin

**Affiliations:** 1 Department of Industrial Design, Chang Gung University, Kweishan, Taoyuan, Taiwan, R.O.C.; 2 Graduate Institute of Rehabilitation Science, Chang Gung University, Kweishan, Taoyuan, Taiwan, R.O.C.; 3 Division of Endocrinology and Metabolism, Department of Internal Medicine, Chang Gung Memorial Hospital, Kweishan, Taoyuan, Taiwan, R.O.C.

**Keywords:** serum uric acid, hyperuricemia, metabolic syndrome, discriminate analysis, cardiovascular risk factor

## Abstract

**Background:**

Metabolic syndrome (MetS) and hyperuricemia are important risk factors for cardiovascular disease. However, findings regarding the relationship between serum uric acid (UA) level and components of MetS have been inconsistent. This study was performed to explore the potential value of UA level as a marker of MetS among male and female Chinese of different ages.

**Methods:**

A total of 5896 subjects (2960 females and 2936 males) were recruited from the Department of Health Management at the Chang Gung Medical Center. Hyperuricemia was defined as a serum UA value >7.0 mg/dL for males or >6.0 mg/dL for females. MetS was defined according to the criteria of the Adult Treatment Panel III, as modified for Chinese subjects. Serum UA was used to differentiate MetS and to calculate epidemiological indices by means of discriminate analysis and logistic regression.

**Results:**

The sensitivity and specificity of serum UA concentration as a marker of MetS ranged from 55.2% to 61.4% and 61.9% to 68.4%, respectively. Subjects with high UA had a higher risk of MetS, with odds ratios ranging from 1.23 to 1.82 (*P* < 0.01). A positive correlation between serum UA and MetS was observed in both sexes. Serum UA and the occurrence of MetS rose with increasing age in females; in males, however, UA values did not vary with age.

**Conclusions:**

Serum UA is more closely associated with MetS in females than in males. High UA among middle-aged women may predict the development of MetS.

## INTRODUCTION

Although the role of hyperuricemia in cardiovascular and renal disease remains controversial, the findings of recent population studies conducted in different parts of the world are consistent with a strong relationship between elevated serum uric acid (UA) levels and the presence of metabolic syndrome (MetS).^1^^–^^5^ Associations between serum UA and specific cardiovascular risk factors, including accumulation of carotid artery plaques and the presence of 1 or more components of MetS, have also been recently reported.^6^^–^^11^ In contrast, in subjects with untreated essential hypertension, no association between serum UA and organ damage was observed,^12^ suggesting that UA is not an independent risk factor for these subjects.

Hyperuricemia and the risk for MetS may vary with age and sex. For example, gouty arthritis and cardiovascular complications are rarely observed in premenopausal females; however, the incidences of hyperuricemia and MetS increase dramatically after menopause.^13^^,^^14^ Use of UA as a surrogate marker of insulin resistance in older females has been recommended.^15^ In studies of white women, sex hormones were associated with the development of MetS and risk factors for the syndrome, including elevated UA.^6^ Evaluation of UA levels with respect to the presence of MetS in men and women of different ages should therefore prove informative.^16^ Although hyperuricemia is generally considered a strong, independent, predictor of MetS in both sexes,^4^ a clear correlation has not been established. Furthermore, it is unclear whether hyperuricemia increases with age or is sex-specific in Chinese. The present study evaluated Taiwanese adolescents and adults for hyperuricemia and risk factors for MetS to ascertain whether direct correlations between these parameters were a function of age and/or sex.

## METHODS

### Participants

Subjects for this investigation were recruited from the Department of Health Examination at the Chang Gung Memorial Hospital in Kweishan, Taoyuan, Taiwan during the period from 2003 through 2005. All subjects in this study were Chinese residents of Taiwan. The study group comprised 5896 subjects, including 2960 females and 2936 males. The average age of the participants was 53.9 ± 12.0 years (range, 17 to 95 years); the average age was 53.5 ± 11.5 years (range, 17 to 95 years) for females and 54.4 ± 12.5 years (range, 18 to 90 years) for males. Because some of subjects recruited from the Department of Health Examination were undergoing examinations for health problems, the authors acknowledge the presence of bias in this study.

Different methods were used to evaluate the association between serum UA and MetS and to differentiate MetS as a function of UA concentration. UA values were used to divide the subjects into subgroups according to sex. The UA values (in mg/dL) for the 10 subgroups of females were 3.8 or lower, 3.9 to 4.3, 4.4 to 4.6, 4.7 to 5.0, 5.1 to 5.3, 5.4 to 5.6, 5.7 to 6.1, 6.2 to 6.6, 6.7 to 7.4, and 7.5 or higher. The corresponding UA values for the 10 subgroups of males were 4.9 or lower, 5.0 to 5.5, 5.6 to 5.9, 6.0 to 6.3, 6.4 to 6.7, 6.8 to 7.1, 7.2 to 7.6, 7.7 to 8.1, 8.2 to 9.1, and 9.2 or higher.

Age is considered the most important factor affecting UA concentration and the development of MetS. Therefore, subjects between 26 and 75 years of age were allocated to subgroups based on 5-year age-bands. Subjects were also divided into 3 standard age groups: young (44 years or younger), middle-aged (45–64 years), and elderly (65 years or older).^17^^,^^18^ The average ages of the young, middle-aged, and elderly groups were 37.0, 53.3, and 70.8 years, respectively, for females and 37.6, 54.0, and 71.4 years, respectively, for males.

### Measurements

Venous blood samples were obtained after an overnight fast and subjected to centrifugation at 3000 rpm for 30 minutes at 4°C. UA present in blood samples was measured by a colorimetric enzymatic method. Hyperuricemia was defined as a UA value above 6.0 mg/dL for males or above 7.0 mg/dL for females.^4^^,^^19^ MetS was defined according to the criteria of the modified Adult Treatment Panel (ATP) III issued in 2004 by the Bureau of Health Promotion, Department of Health, ROC (Taiwan). Subjects satisfying 3 or more of the following criteria were defined as having MetS: central obesity with a waist circumference >90 cm for males or >80 cm for females, or BMI ≥27 kg/m^2^; triglyceride ≥150 mg/dL (1.695 mmol/L); high-density lipoprotein (HDL) cholesterol <40 mg/dL (1.036 mmol/L) for males or <50 mg/dL (1.295 mmol/l) for females; blood pressure ≥130/85 mm Hg; and fasting glucose ≥100 mg/dL (≥5.6 mmol/L).^19^ The study was approved by the Institutional Review Board of Chang Gung Memorial Hospital. Subjects with an obvious disease or disorder, or a weight change exceeding 10%, during the 6 months before initiation of the study were excluded.

### Statistical analysis

All data analyses were performed using SPSS software. Separate statistical analyses were performed for males and females and for each age group. Normal variables were evaluated by using the chi-square test for the presence or absence of MetS and hyperuricemia. Discriminate analysis was used to test the correlation between MetS and UA. In addition, serum UA concentration was utilized to distinguish MetS and to calculate sensitivity, specificity, pre-test probability (prevalence), positive likelihood ratio (LR+), and negative likelihood ratio (LR−). The following formulas were used to calculate the parameters relevant to this analysis (a: subject with MetS who has been identified as positive; b: subject without MetS who has been identified as positive; c: subject with MetS who has been identified as negative; d: subject without MetS who has been identified as negative): sensitivity = a/(a + c); specificity = d/(b + d); LR+ = sensitivity/(1 − specificity); LR− = (1 − sensitivity)/specificity; pre-test probability (prevalence) = (a + c)/(a + b + c + d); and post-test probability = post-test odds/(post-test odds + 1), with post-test odds = [a/(a + b)] * [prevalence/(1 − prevalence)].^17^ Logistic regression was used to analyze the odds ratio (OR) of UA by using the presence of MetS as the dependent variable. *P* values <0.05 were considered statistically significant.

## RESULTS

**Figure 1.**
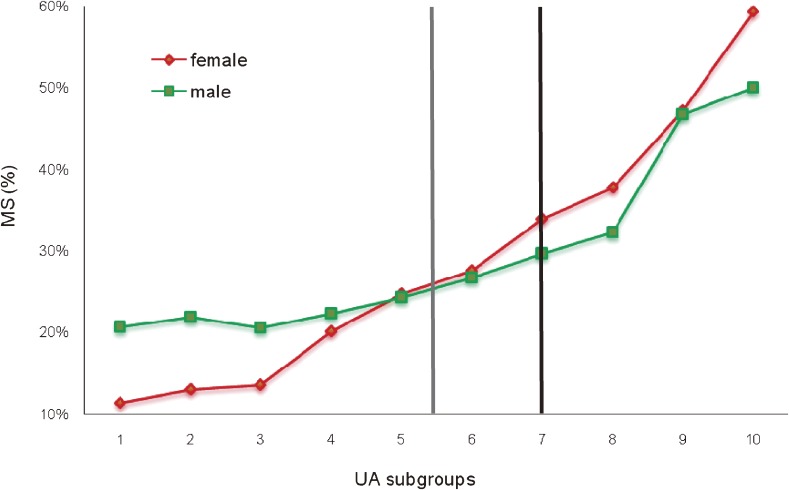
Percentages of female and male subjects with metabolic syndrome (MetS), by subgroup of serum uric acid (UA) concentration.

The percentages of female and male subjects with MetS in each of the 10 subgroups of UA concentration are shown in Figure 1. For the 3 subgroups with the lowest UA concentrations, the proportion of those with MetS was below 15% for females and approximately 20% for males. Increases in the percentages of subjects with MetS were observed from the fourth through the 10th subgroup of UA concentration, with a sharp rise in the slope of the increment in females. The percentage of female subjects with MetS was higher than that of males for the fifth through the 10th subgroup of UA concentration. The gray and black vertical lines in Figure 1 show the average serum UA value for females and males, respectively. At the “crossover” point for the 2 plots, UA concentrations ranged from 5.1 to 5.6 mg/dL for females and from 6.4 to 7.1 mg/dL for males. Hyperuricemia was defined as a UA of 6.0 mg/dL for females or 7.0 mg/dL for males. Thus, it is clear from Figure 1 that the rate of occurrence of MetS is higher in subjects with hyperuricemia as compared with those without it. In the subgroup with the highest UA, 59.4% of female subjects and 50% of male subjects had MetS.

**Figure 2.**
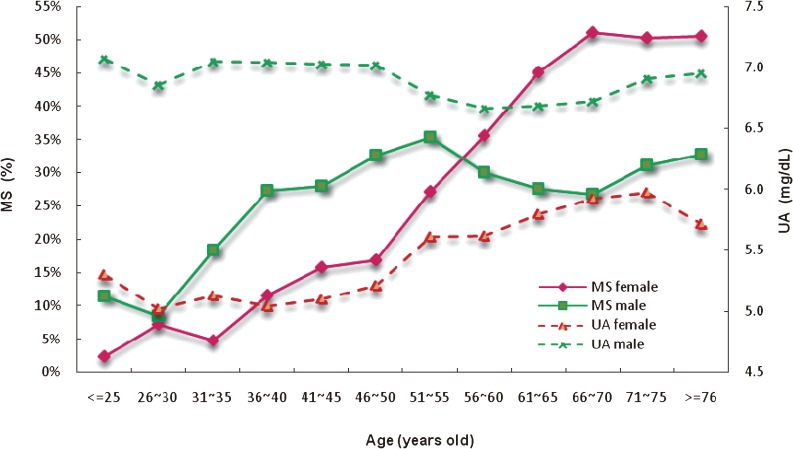
Percentages of female and male subjects with metabolic syndrome (MetS), and serum uric acid (UA) levels in female and male subjects, by 5-year age-band.

Figure 2 shows the percentages of female and male subjects with MetS and hyperuricemia by 5-year age band. UA values for males were clustered around 7.0 mg/dL; the highest concentration (7.1 mg/dL) was observed at 25 years of age or younger and the lowest concentration (6.7 mg/dL) at 56 to 60 years of age. For females, serum UA values increased with age, and the peak value (6.0 mg/dL) was observed at age 71 to 75 years. The percentage of female subjects with MetS increased with age and reached a plateau at age 66 to 70 years. The percentage of male subjects with MetS also rose with increasing age until age 55 years; at this age, 35.4% of males had developed MetS. After age 55 years, however, the percentage of subjects with MetS slightly decreased with increasing age in males but continued to rise with increasing age in females. In addition, after age 55 years, UA concentration and the percentage of subjects with MetS tended to be parallel regardless of sex.

We further analyzed the effects of age on MetS and serum UA concentration by reducing the age groups to 3—young, middle-aged, and elderly subjects—for both females and males. These groups were then divided into subgroups based on the absence or presence of hyperuricemia or MetS (Table 1). Significant differences were observed as a function of age and MetS and as a function of age and hyperuricemia (*P* < 0.05), except for the category of male subjects with or without hyperuricemia. These findings indicate that among these 3 age groups, the distributions of both MetS and hyperuricemia differ significantly. Of the 631 male subjects in the young group, the 1652 in the middle-aged group, and the 653 in the elderly group, 146 (23.1%), 524 (31.7%), and 194 (29.7%), respectively, satisfied the criteria for MetS. In females, age was a major factor influencing MetS: 57 (10.8%), 521 (27.3%), and 262 (50%) of females had MetS in the young, middle-aged, and elderly groups, respectively. Age and sex differences were also observed when the other parameter, hyperuricemia, was examined. A total of 119 (22.5%) females and 293 (46.4%) males in the young group, 622 (32.6%) females and 720 (43.6%) males in the middle-aged group, and 226 (43.1%) females and 282 (43.2%) males in the elderly group had hyperuricemia. Although the incidence of hyperuricemia, like that of MetS, differed with age and sex, the incidence was higher for male subjects than for female subjects in the young and middle-aged groups.

**Table 1.** Numbers and percentages of subjects with MetS (and its components) and hyperuricemia, by sex and age

MetS or hyperuricemia	Sex:	Female				Male			
		
	Age:	Young	Middle	Old	*P*	Young	Middle	Old	*P*
Non-MetS		472	1386	262		485	1128	459	
MetS		57	521	262	<0.001	146	524	194	<0.001
MetS rate		10.8%	27.3%	50.0%		23.1%	31.7%	29.7%	
Central obesity^a^		36.7%	62.9%	84.9%	<0.001	39.6%	50.1%	52.8%	<0.001
Hypertension^a^		25.3%	45.5%	66.4%	<0.001	46.9%	59.4%	56.7%	<0.001
Hyperglycemia^a^		3.6%	13.0%	25.6%	<0.001	6.7%	18.3%	22.2%	<0.001
Hypertriglyceridemia^a^		11.2%	24.8%	38.2%	<0.001	34.9%	35.8%	27.7%	<0.001
Low HDL^a^		20.4%	28.4%	37.2%	<0.001	21.4%	22.6%	26.2%	0.094
		
		Young	Middle	Old	*P*	Young	Middle	Old	*P*
		
Normal UA		410	1285	298		338	932	371	
Hyperuricemia		119	622	226	<0.001	293	720	282	0.408
Hyperuricemia		22.5%	32.6%	43.1%		46.4%	43.6%	43.2%	

^a^Five components of MetS.

Abbreviations: MetS, metabolic syndrome; HDL, high-density lipoprotein; UA, serum uric acid.

Table 2 shows the odds ratios of MetS in terms of serum uric acid concentration for all subjects. The highest risk was observed in middle-aged women (OR: 1.82; 95% confidence interval [CI]: 1.65–2.00). The lowest risk among females (OR: 1.43; 95% CI: 1.32–1.55) was higher than the highest risk (OR: 1.38; 95% CI: 1.10–1.74) among males. Regarding sex-specific rates, the lowest risks were observed in elderly women and elderly men. The risk of MetS in terms of high serum uric acid concentration was statistically significant in both males and females in all age groups, except for young females. However, in both female and male subjects, the coefficients of correlation between MetS and serum UA concentration were positive. For male subjects, this coefficient ranged from 0.18 to 0.25 (*P* < 0.01 for all) and was highest for young males. Among females, this coefficient ranged from 0.25 to 0.32 (*P* < 0.01 for all) and was highest for middle-aged women.

**Table 2.** Epidemiological variables and odds ratios for MetS, by sex and age group

	Sex:	Female			Male		
			
	Age:	Young	Middle	Old	Young	Middle	Old
Odds ratio ​ (95% CI)		1.73 (0.95–3.14)	1.82^a^ (1.65–2.00)	1.43^a^ (1.32–1.55)	1.38^a^ (1.10–1.74)	1.32^a^ (1.22–1.42)	1.23^a^ (1.19–1.37)
Correlation		0.26^a^	0.32^a^	0.25^a^	0.25^a^	0.21^a^	0.18^a^
Sensitivity		60.2%	61.4%	58.0%	58.9%	55.9%	55.2%
Specificity		66.1%	68.4%	65.3%	66.0%	62.2%	61.9%
LR (+)		1.88	1.94	1.67	1.73	1.48	1.45
LR (−)		0.59	0.56	0.64	0.62	0.71	0.72
Pre-test ​ probability		10.8%	27.3%	50.0%	23.1%	31.7%	29.7%
Post-test ​ probability		3.1%	13.7%	38.5%	9.4%	15.9%	13.8%

^a^*P* < 0.01.

Abbreviations: MetS, metabolic syndrome; LR (+), positive likelihood ratio; LR (−), negative likelihood ratio

The sensitivity of serum UA concentration for the diagnosis of MetS ranged from 58.0% to 61.4% in females and from 55.2% to 58.9% in males. The highest specificities were observed in young females and young males—68.4% and 66.0%, respectively. The lowest specificities were observed in elderly subjects—65.3% for women and 61.9% for men. In both sexes, the likelihood ratios for a positive test result were higher in the young and middle-aged groups than in the elderly group, especially for young females (1.94). For males, the likelihood ratios for a negative test result were similar in all 3 age groups and ranged from 0.62 to 0.72; for females, these ratios ranged from 0.56 to 0.64. Regarding pre-test probabilities, this index rose sharply with increasing age for females with MetS: 10.8%, 27.3%, and 50.0% for young, middle-aged, and elderly subjects, respectively. In male subjects, the highest prevalence was 31.7% in the middle-aged group and the lowest was 23.1% in the young group. Post-test probabilities, however, were lower than prevalences in all populations. The post-test probability for high serum UA concentration in the identification of MetS was almost 38.5% in elderly females, and the deviation between prevalence and post-test probability indices ranged from 7.7% to 15.9%.

## DISCUSSION

This study was performed to evaluate serum UA concentration as a marker of MetS among Chinese males and females of different ages. We found that high UA was associated with an increased risk of MetS.^3^^,^^18^ However, UA concentration was more closely associated with MetS in females than in males,^16^ and the highest risk of MetS was observed in middle-aged women with high UA.

These findings are in good agreement with those of previous studies. In this study, as in a recent study of elderly Taiwanese,^18^ the prevalence of hyperuricemia was found to be significantly higher among subjects with MetS, as compared with those without MetS. High UA has also been found to correlate with several risk factors for MetS^19^; in adult populations, elevated serum UA was independently correlated with these risk factors.^10^ Furthermore, UA was found to rise as the number of components of MetS in a person increased.^20^ Sex-specific distribution of the occurrence of MetS among subjects with different serum UA concentrations has also been observed. In a prospective analysis of the predictive value of hyperuricemia in residents of the United States,^4^ males with a serum UA value ≥6.5 mg/dL were found to have a 1.60-fold increased risk of MetS as compared to males with a concentration <5.5 mg/dL; in females with a serum UA concentration ≥4.6 mg/dL, the risk was at least 2-fold higher. In recent studies describing an association between hyperuricemia and the risk of MetS, the odds ratio was found to be similar for males and females when hyperuricemia was defined as a UA level of 6 mg/dL for females and 7 mg/dL for males.^21^ Lastly, a close association of hyperuricemia with MetS has been observed among different ethnic groups.^2^^,^^3^^,^^5^

Because the incidence of MetS was relatively low in females and males with low UA, a UA value below the average represents a low risk for MetS. A UA value higher than average but below the cutoff value for hyperuricemia should be considered medium risk for MetS. In the present study, the incidence of MetS was actually much higher among subjects in the medium-risk category than among those in the low-risk category. A UA value in the hyperuricemic range should be regarded as high-risk because the incidence of MetS was markedly higher at these concentrations, especially in females.

Sex is clearly an important factor in the inter-relationships among hyperuricemia, MetS, and age. A limitation of the present study was the absence of information on the menopausal status of women. However, in other investigations, the rates of MetS and hyperuricemia were consistently higher in women of postmenopausal age than in premenopausal women.^19^^,^^22^^,^^23^ In the present study, the most important observation regarding sex was that the incidence of MetS in females with lower-than-average UA was significantly lower than that in males with the same UA values. Overall, the findings of the present study agree with those of a recent study^5^ in which serum UA was found to be associated with metabolic syndrome and some of its components in Japanese. In that study, however, rates of hyperuricemia were similar in elderly men and women, but women with hyperuricemia had higher rates of MetS than did men with hyperuricemia.

Analyses examining both sex and age revealed that the incidence of MetS differed between men and women aged 51 to 55 years and 56 to 60 years, and that the age of 55 years was found to be critical for women and men when these subjects were grouped by 5-year increments. Other findings indicate that sex significantly influences serum UA and the occurrence of MetS during aging.^19^^,^^22^^,^^23^

Additional analysis disclosed that the distribution of male subjects with or without hyperuricemia did not differ among the 3 major age groups. This result is at odds with a previous study, which showed that serum UA rose with increasing age.^14^ This discrepancy may be due to differences in lifestyle, eating habits, and health status among the subjects of these studies.^24^ In the present study, the percentages of subjects with MetS and with hyperuricemia rose significantly in middle-aged and elderly women, but not in men, most likely because MetS and its components develop more frequently after the onset of menopause.^22^ Indeed, as noted above, postmenopausal women have consistently higher rates of MetS and hyperuricemia than do premenopausal women.^13^^,^^18^

This study is the first to use discriminate analysis to examine the association between MetS and serum UA concentration in males and females in different age groups. The post-test odds of MetS were determined by the likelihood ratio, the prevalence of MetS, the characteristics of the subject pool, and information regarding individual subjects. Identification of MetS by serum UA was better in females than in males. Moreover, the coefficient of correlation was higher in females than in males.

In conclusion, serum UA values differ significantly with age and sex in Chinese. Serum UA concentration correlates positively with MetS in these subjects, and is more closely associated with MetS in females than in males. These findings provide a very useful predictor of MetS in middle-aged women. Further prospective studies that investigate the mechanism(s) by which sex influences the correlation between serum UA and MetS are recommended.
